# DeepGAM: An interpretable deep neural network using generalized additive model for depression diagnosis: Data from the heart and soul study

**DOI:** 10.1371/journal.pone.0324169

**Published:** 2025-09-05

**Authors:** Chiyoung Lee, Yeri Kim, Seoyoung Kim, Mary Whooley, Heewon Kim

**Affiliations:** 1 The University of Arizona College of Nursing, Tucson, Arizona, United States of America; 2 Global School of Media, College of IT, Soongsil University, Seoul, Korea; 3 School of Medicine, University of California - San Francisco, California, United States of America; Majmaah University, SAUDI ARABIA

## Abstract

Deep neural networks have achieved significant performance breakthroughs across a range of tasks. For diagnosing depression, there has been increasing attention on estimating depression status from personal medical data. However, the neural networks often act as black boxes, making it difficult to discern the individual effects of each input component. To alleviate this problem, we proposed a deep-learning-based generalized additive model called DeepGAM to improve the interpretability of depression diagnosis. We utilized the baseline cross-sectional data from the Heart and Soul Study to achieve our study’s aim. DeepGAM incorporates additive functions based on a neural network that learns to discern the positive and negative impacts of the values of individual components. The network architecture and the objective function are designed to constrain and regularize the output values for interpretability. Moreover, we used a direct-through estimator (STE) to select important features using gradient descent. The STE enables machine learning models to maintain their performance using a few features and interpretable function visualizations. DeepGAM achieved the highest AUC (0.600) and F1-score (0.387), outperforming neural networks and IGANN. The five features selected via STE performed comparably to 99 features and surpassed traditional methods such as Lasso and Boruta. Additionally, analyses highlighted DeepGAM’s interpretability and performance on public datasets. In conclusion, DeepGAM with STE demonstrated accurate and interpretable performance in predicting depression compared to existing machine learning methods.

## Introduction

Depression is a disorder of major public health importance, given its prevalence and the associated suffering, dysfunction, morbidity, and economic burden [1]. The Global Burden of Disease report estimates that the point prevalence of unipolar depressive episodes is 1.9% for men and 3.2% for women, while the one-year prevalence is estimated to be 5.8% for men and 9.5% for women. If current trends in demographic and epidemiological transitions continue, the burden of depression is projected to increase to 5.7% of the total burden of disease by 2020 [1]. Detecting depression, therefore, represents a critical public health challenge. In this study, we utilized baseline data from the Heart and Soul Study [2]—a prospective cohort study examining the impact of psychosocial factors on the prognosis of stable coronary heart disease (CHD)—to estimate patients’ depression status by analyzing a range of psychosocial and behavioral factors, as well as objective biomarkers.

Recent advances in machine learning (ML) have lead to breakthroughs in various disease diagnosis tasks [3–6]. These ML models capture complex patient medical data patterns and identify important disease features. In particular, recent studies expand the application of ML and deep learning methods for predicting and diagnosing depression, a socially concerning mental disorder [7–9]. However, deep neural networks are often regarded as “black boxes,” making it challenging to interpret how decisions are made internally.

This study evaluated the performance of various machine learning techniques for diagnosing depression using the Heart and Soul Study dataset. The primary hypothesis of this study is that incorporating the neural networks, the Generalized Additive Model (GAM), and the straight-through estimator (STE) technique can achieve more interpretable results for depression diagnosis compared to conventional machine learning techniques and feature selection methods, while also maintaining similar performance even with a reduced number of features. To this end, we utilized an activation function to limit maximum influences and a regularizer to minimize average influences, facilitating a better understanding of feature effects. We compared the performance of various machine learning models and feature selection methods using performance metrics such as AUC, accuracy, F1-score, precision, recall, and specificity.

The experimental results demonstrated that the proposed model, DeepGAM, outperformed machine learning methods, such as SVM, neural networks, and IGANN, in diagnosing depression. Additionally, STE discovered more effective features for diagnosis than traditional feature selection methods, such as Lasso and Boruta. DeepGAM and STE visualized the positive and negative effects of five selected features on depression, and we further analyzed the impact of limiting maximum influence and minimizing average influence to enhance the interpretability of DeepGAM.

This study is significant because it enables interpretable depression diagnosis compared to traditional ML techniques. Unlike traditional models, where it is difficult to understand how each factor contributes to depression, it allows a clear interpretation of the influence of each predictor variable. This characteristic provides clinically valuable information, improving accuracy and transparency in depression diagnosis.

Our contributions are as follows:

- We introduced DeepGAM, a model that employs neural networks for generalized additive models, enabling the interpretation of the impact of individual medical components on depression.
- We utilized a differentiable feature selection approach using a straight-through estimator (STE), enabling DeepGAM to maintain diagnostic performance with only five features.
- Experiments presented the performance of depression diagnosis across different machine learning methods using the Heart and Soul Study dataset.
- Analyses examined the impact of each technique utilized in DeepGAM for enhancing interpretability.

## Related works

### Machine learning models for disease diagnosis

In recent medical research, machine learning (ML) has emerged as a crucial technique significantly enhancing the performance of disease diagnosis. Considering the high cost and privacy concerns of collecting medical data, this research typically concentrates on specialized datasets. It employs conventional machine learning techniques such as Support Vector Machine (SVM), AdaBoost, logistic regression, random forest, and neural networks. Convolutional neural networks and transformers are utilized for high dimensional data (e.g., medical images). ML approaches have been vital for disease diagnosis, patient classification, and prognosis in recent cancer research and medical oncology [3], clinical epigenetics [4], heart failure [6], heart disease [10], and Alzheimer’s disease based on multimodal neuroimaging data [5].

In depression diagnosis, various machine-learning approaches have been applied to enhance predictive accuracy. Depression, in particular, has had a high prevalence rate among cardiac patients and myocardial infarction patients [11–13]. Additionally, depression is associated with blood pressure [14,15] and biomarkers [16]. Zhang et al. utilized the CatBoost algorithm to develop a depression prediction model for middle-aged and elderly populations [17], while Haque et al. adopted machine learning techniques for detecting childhood depression. Lee and Kim focused on diagnosing depression within hypertensive populations using ML methods [9]. Similarly, Mao et al. improved automated depression diagnosis accuracy through a multimodal approach combining voice and text data [18]. Furthermore, Aleem et al. compared the performance of various machine learning algorithms for depression diagnosis [19], and Bhadra et al. conducted a systematic review of ML-based depression diagnosis research [20]. Squires et al. analyzed the contributions of deep learning and machine learning in detecting, diagnosing, and treating depression within the field of psychiatry [21]. However, neural networks, while powerful, face limitations in interpretability, making them challenging to employ as supplementary tools for diagnosing complex depression cases.

### Interpretable neural networks

Interpretable neural networks are a crucial research area that enhances the understanding and trustworthiness of model decision-making processes. However, the term interpretability is not well-defined and often carries different meanings in various studies [22]. LIME [23] provides explanations for the predictions of any classifier in an interpretable and faithful manner. DeepLIFT [24] offers fine-grained insights into model decisions by comparing the activation of each neuron and assigning contribution scores accordingly. SHAP [25] integrates game theory with local explanations and provides a unified measure of feature importance. SENN [26] presents the inherent interpretability of model architectures, which is understandable to humans. ICNN [27] modified traditional convolutional neural networks into interpretable through each filter representing a certain object part. Recently, IGANN [28] introduced a neural network based on a generalized additive model. IGANN has been the closest in similarity to our DeepGAM. However, DeepGAM enhances both its representational power and interpretability by improving network architectures and loss functions, and we utilized an effective feature selection method that can be applied during DeepGAM training.

### Generalized additive models in medical data analysis

The Generalized Additive Model (GAM) is a flexible model framework that combines the outputs of non-linear functions from each input, enabling the visualization of each component’s impact and facilitating effective analysis of medical data [29]. In medical applications, GAMs have been widely used for various types of data analysis. For example, Clements et al. used GAMs to predict lung cancer incidence [30], and Mukherjee et al. applied GAMs to identify diagnostic and risk factors in heart disease patients [31]. Papoila et al. compared the performance of GAMs and Generalized Linear Models (GLMs) on medical data to assess how each performs under different conditions [32]. Similarly, Zhang et al. [33] examined the relationship between altitude and hypertension prevalence, modeling non-linear relationships in the data effectively using GAMs. He et al. [34] analyzed the relationship between COVID-19 cases and climatic variables, where GAMs were applied to time-series data to predict changes in infection rates. Rostami et al. [35] used GAMs to determine optimal vitamin D cutoff points concerning pregnancy outcomes, supporting maternal health management, and Vieira et al. employed GAMs for spatial analysis of bladder, kidney, and pancreatic cancers in Cape Cod [36]. In hematology, Lan et al. [37] utilized GAMs to analyze lymphoma patient data for diagnosis and treatment. Here, we adopted DeepGAM for depression diagnosis and analyze the impact of each component.

## Depression dataset and methods

### Dataset and sample

This study used baseline cross-sectional data from the Heart and Soul Study. The Heart and Soul Study is a prospective cohort study originally designed to investigate how psychological disorders lead to CHD events in outpatients with stable CHD. This study used baseline cross-sectional data from the Heart and Soul Study. The Heart and Soul Study is a prospective cohort study originally designed to investigate how psychological disorders lead to CHD events in outpatients with stable CHD. The enrollment process and methods were previously described in another study [2]. Briefly, patients were recruited from clinics at the San Francisco Veterans Affairs (VA) Medical Center, the Palo Alto VA Health Care System, the University of California San Francisco Medical Center, and the San Francisco Community Health Network. Patients were considered eligible for study participation if they fulfilled at least one of the following criteria: a history of myocardial infarction (MI), angiographic evidence of at least 50% stenosis in one or more coronary vessels, previous evidence of exercise-induced ischemia using treadmill or nuclear testing, or a history of coronary revascularization. Exclusion criteria included a history of myocardial infarction in the past 6 months, self-perceived inability to walk for a distance of one block, or having plans to move out of the local area within 3 years.

From September 2000 to December 2002, 1,024 patients enrolled in the study, whose samples were analyzed. The participants were 60% non-Hispanic white, 9% Hispanic white, 17% African American, 11% Asian, and 3% other. The mean age was 67 (range 45-90) years at baseline.

### Ethics statement

For the present secondary data analysis, the first author received the data from the principal investigator of the Heart and Soul Study after receiving approval from the University of Arizona. All methods and procedures were conducted in accordance with the relevant guidelines and regulations. The Heart and Soul Study was approved by the following institutional review boards: the Committee on Human Research at the University of California, San Francisco; the Research and Development Committee at the San Francisco Veterans Affairs Medical Center; the Medical Human Subjects Committee at Stanford University; the Human Subjects Committee at the Veterans Affairs Palo Alto Health Care System; and the Data Governance Board of the Community Health Network of San Francisco. Participants provided written informed consent prior to their enrollment.

### Measurement at the baseline examination

#### Target of ML models: Depression.

The Heart and Soul Study assessed the presence of major depression within the past month with the Computerized National Institute of Mental Health Diagnostic Interview Schedule (CDIS-IV). The CDIS-IV is a highly structured interview designed to yield psychiatric diagnoses according to DSM-IV (Diagnostic and Statistical Manual of Mental Disorders, 4th Edition) criteria [38]. Trained research assistants administered the interview during the daylong study appointment.

#### Input of ML models.

Comprehensive baseline data for each subject were collected at enrollment. Demographic factors (e.g., age, ethnicity, gender), lifestyle variables (e.g., alcohol use, smoking status, physical activity), and medical history were assessed via questionnaire. A 12-hour fasting blood sample was obtained during the morning of the enrollment visit. Participants also collected 24-hour urine specimens for the measurement of creatinine, free cortisol, and catecholamines. Samples of serum, plasma, DNA, and 24-hour urine remain stored at –80 degrees Celsius. Examples of laboratory tests include C-reactive protein (mg/dl), pentraxin 3 (ng/ml), hemoglobin A1c (%), plasma dopamine (pg/ml), folic acid (ng/ml), telomere length, urine creatinine (mg/dl), urine calcium (mg/ml), urinary norepinephrine (mcg/day), and many others. All variables were carefully selected based on evidence from the literature demonstrating their established association with depression [39,40].

### Feature selection using Straight-Through Estimator (STE)

Considering that not all features hold meaningful information for depression, we conducted a feature selection method to eliminate redundant features for model performance. We utilized a differentiable straight-through estimator (STE) [41] to find important features by gradient descent. The STE facilitates end-to-end training: simultaneous feature selection and model parameter training. Such holistic training helps to find important features for model parameters that change during training. Formally,

$$g(\phi )=\left\{ \begin{array}{cc} \text{round}(\sigma (\phi )) & \text{if Forward pass}\\ \sigma (\phi ) & \text{if Backward pass}, \end{array}\right.$$ (1)

where *ϕ* is a learnable parameter and σ(ϕ) denotes $\frac{1}{1+e^{-\phi }}$, a sigmoid function of *ϕ*.

We aimed to select features with a significant influence on depression. The sigmoid function represents the influence of a feature between 0 and 1. If the influence does not surpass a set threshold (0.5), the feature value is multiplied by 0 as “dead” to ensure it doesn’t affect subsequent learning. The number of “alive” features was progressively reduced during training. We set training hyperparameters for approximately ten ‘alive’ features at the end of the training.

### DeepGAM

We used the dataset consisting of *N* patients with depression labels, represented as $\left\{(x_{1},y_{1}),(x_{2},y_{2}),\ldots ,(x_{N},y_{N})\right\}$. Here, $x_{i}$ represents the features of the *i*-th patient (e.g., dopamine, leptin, insulin, etc.), and *y*_*i*_ denotes the label of the patient, i.e., whether they are diagnosed with major depression *y*_*i*_ =  + 1 or not *y*_*i*_ = −1. To simplify the expression, we did not use the notation *i* unless it is specifically mentioned.

**Fig 1:**
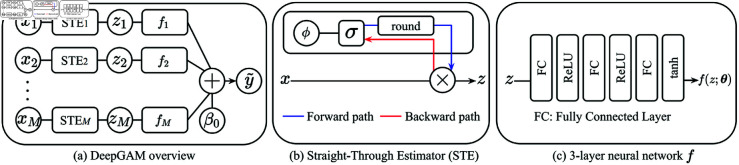
Network architecture of DeepGAM. ( **a**) Each feature $x_{j}\in {\mathbb{R}}^{1}$ passes through a Straight-Through Estimator (STE), which learns to select relevant features during training. The selected feature *z*_*j*_ is then fed into a 3-layer neural network *f*, and and its output is combined with a learnable bias parameter $\beta_{0}$ to represent the depression status. ( **b**) The STE selects a feature by multiplying it with a binary value in the forward pass, and enables parameter *ϕ* to be updated via gradient descent by omitting the non-differentiable round function in the backward pass. ( **c**) The 3-layer neural network consists of Fully Connected (FC) layers with ReLU and tanh activations. Each feature has distinct learnable parameters *ϕ* and θ.

Our model predicted a scalar value and determined the depression status by thresholding; if the output value is greater than the threshold *t*, it returns 1 (depression), and otherwise, it returns –1 (normal). To interpret the effect of each feature on depression, we adopted a Generalized Additive Model (GAM) framework using neural networks as described in Fig 1. The rest of this section describes the GAM framework, neural network design, and learning objectives.

#### Generalized Additive Model (GAM) framework.

GAM [29] is a nonlinear additive function of the independent variables (or features), which is an extension of the Generalized Linear Model (GLM) by replacing the linear additive term in GLM with a more general additive term. Given an input $x\in {\mathbb{R}}^{M}$ with *M* features and a predicted value $\tilde{y}\in \mathbb{R}$, for each feature j∈{1,2,...,M}, the GLM can be expressed as

$$\tilde{y}=\beta_{0}+\beta_{1}x_{1}+\beta_{2}x_{2}+\ldots +\beta_{M}x_{M},$$ (2)

where $\beta_{j}\in \mathbb{R}$ is a hyperparameter, and GAM can be expressed as

$$\tilde{y}=\beta_{0}+f_{1}(x_{1})+f_{2}(x_{2})+\ldots +f_{M}(x_{M}),$$ (3)

Where *f*_*i*_ is a general nonlinear function. Finally, we formulated *f*_*j*_ as the *j*-th neural network parameterized by $\theta_{j}$ and used the selected feature *z*_*j*_ as input:

$$\tilde{y}=\beta_{0}+f_{1}(z_{1};\theta_{1})+f_{2}(z_{2};\theta_{2})+\ldots +f_{M}(z_{M};\theta_{M}).$$ (4)

where $z_{j}=x_{j}\;\cdot \;g(\phi_{j})$ and $g(\phi_{j})$ is a binary value in the forward pass described in (Eq 1), selecting the *j*-th feature with the value of 1. We omitted the notation *j* for simplicity unless specified otherwise.

#### Neural network architecture.

We used the PyTorch library for the 3-layer neural network with ReLU and tanh activations as a modeling function (*f*) to predict a scalar from a feature. With the feature selection method in (Eq 1), the neural network formally becomes:

$$f(z;\theta )=\tanh(\theta^{\left(3\right)}\max(0,\theta^{\left(2\right)}\max(0,\theta^{\left(1\right)}z))),$$ (5)

where $\theta^{\left(1\right)}\in {\mathbb{R}}^{C\times 1}$, $\theta^{\left(2\right)}\in {\mathbb{R}}^{C\times C}$, $\theta^{\left(3\right)}\in {\mathbb{R}}^{1\times C}$ are the per-layer weights of the neural network with *C* channels. The tanh activation restricts the output to values between –1 and 1, which prevents undue influence from a feature.

#### Loss function.

We aimed to learn the parameters of neural networks θ and STE *ϕ* for accurate depression classification with important selected features. Formally, the objective function becomes:

$$\min_{\theta ,\phi }𝔼[ℒ(\tilde{y},y)+\lambda_{1}{ℛ}_{1}(\tilde{y})+\lambda_{2}{ℛ}_{2}(\phi )]$$ (6)

where ℒ is an MSE loss for binary classification and ${ℛ}_{1}$ and ${ℛ}_{2}$ are regularization terms, ${ℛ}_{1}(\tilde{y})=\sum_{j}||f_{j}(z_{j})|{|}^{2}$ and ${ℛ}_{2}(\phi )=\sum_{j}g(\phi_{j})$. $\lambda_{1}$ and $\lambda_{2}$ are hyperparameters to balance three terms. ${ℛ}_{1}$ is an interpretability regularizer that forces to minimize the effect from each feature, preventing a meaningless sum; for instance, one feature predicts +1 and the other feature predicts –1 for all inputs. ${ℛ}_{2}$ is a resource regularizer that forces to minimize the number of “alive” features.

#### Data imbalance management.

The Heart and Soul Study dataset [2] had a class imbalance where depression patients accounted for 22.6% of the total. Machine learning approaches often learn a bias to predict the dominant classes in the training dataset. We used the Imbalanced Dataset Sampler (available at: https://github.com/ufoym/imbalanced-dataset-sampler), which equalizes the class distribution in training by adjusting the sampling ratios for each class.

## Experiments

### Implementation details

#### Measures.

Various performance metrics are utilized based on the confusion matrix for fair performance comparisons of classification methods. The confusion matrix maps the actual classes to the predicted classes. True Positive (TP) denotes instances where depressed patients are predicted as depressed, and True Negative (TN) refers to instances where normal patients are correctly identified as normal. Conversely, False Positive (FP) occurs when normal patients are incorrectly identified as depressed, and False Negative (FN) happens when depressed patients are incorrectly identified as normal.

We employed the measures of Accuracy, F1-score, Precision, Recall, and Specificity, which are defined as follows:


$$\text{Accuracy}=\frac{TP+TN}{TP+TN+FP+FN}$$

$$\text{F1-score}=2\cdot \frac{\text{Precision}\cdot \text{Recall}}{\text{Precision}+\text{Recall}}$$

$$\text{Precision}=\frac{TP}{TP+FP}$$

$$\text{Recall}=\frac{TP}{TP+FN}$$

$$\text{Specificity}=\frac{TN}{TN+FP}$$

We also reported Receiver Operating Characteristic (ROC) curves, plotting the True Positive Rate (TPR) against the False Positive Rate (FPR) at various threshold settings, and Area Under the Curve (AUC) scores with a higher value indicating better model performance.

In regression-based performance comparisons, Mean Squared Error (MSE) and R-squared (${\text{R}}^{2}$) scores were commonly used metrics. MSE is the mean of the squared differences between predicted and actual values, serving as a key measure of prediction error. A lower MSE indicates that the predicted values are closer to the actual values, reflecting higher model performance. The ${\text{R}}^{2}$ score represents how well the model explains the variance in the data, with values closer to 1 indicating a better fit to the data’s variability. Notably, the ${\text{R}}^{2}$ score can also take negative values, signifying that the model performs worse than a simple mean-based prediction, indicating poor explanatory power. The formulas for calculating MSE and ${\text{R}}^{2}$ score are as follows:

$$\text{MSE}=\frac{1}{n}\sum_{i=1}^{n}(y_{i}-{\hat{y}}_{i}{)}^{2}$$ (7)

$$R^{2}=1-\frac{\sum_{i=1}^{n}(y_{i}-{\hat{y}}_{i}{)}^{2}}{\sum_{i=1}^{n}(y_{i}-\bar{y}{)}^{2}}$$ (8)

#### Training hyperparameters.

We employed a Stochastic Gradient Descent (SGD) optimizer with a learning rate of 0.01, a batch size of 16, and 40 epochs. The hyperparameters *C*, $\lambda_{1}$, and $\lambda_{2}$ were set to 128, 1, and 0.005, respectively. The learnable parameters *ϕ* for feature selection were initialized by 0.05. We adopted an L2 regularizer with a weight of 0.001.

To tune these hyperparameters, we used a validation set split from one fold of the training data during cross-validation. Specifically, we performed 5-fold cross-validation to evaluate final performance, and one fold’s training set was further divided into a smaller validation set for hyperparameter tuning. This procedure was designed to prevent information leakage from the test fold during tuning.

For hyperparameter search, we varied each value across a scale ranging from 0.001× to 1000× (e.g., multiplying or dividing by factors of 2 or 10). Our proposed method was generally robust to hyperparameter changes, showing stable performance across a wide range. The selected hyperparameters were also successfully applied to public datasets without significant modification, further demonstrating the robustness of our approach.

#### Machine learning methods to compare.

We compared DeepGAM with Support Vector Machine (SVM), Logistic Regression, and Neural Network. SVM classifies data by finding the hyperplane that best separates the data points of different classes. The points closest to the hyperplane are called support vectors, which support defining the decision boundary. We utilized SVM by a single-layer neural network with margin-based loss. Logistic Regression also employed the single-layer neural network to model the class probability with cross-entropy loss. The Neural Network adopted three fully connected layers with ReLU activation and cross-entropy loss. All training hyperparameters of these methods, except the architectures and loss functions, were identical to DeepGAM for fair comparisons.

#### Feature selection methods to compare.

The proposed feature selection method, denoted as STE, was compared to Lasso(Least Absolute Shrinkage and Selection Operator) [42] and Boruta [43]. Lasso utilizes a linear regression with L1 regularization and selects the important features by the magnitude of the coefficients. Boruta selects features based on the random forest by iteratively comparing the feature importance with randomly permuted copies.

### Results

#### Model performance comparisons.

**Fig 2:**
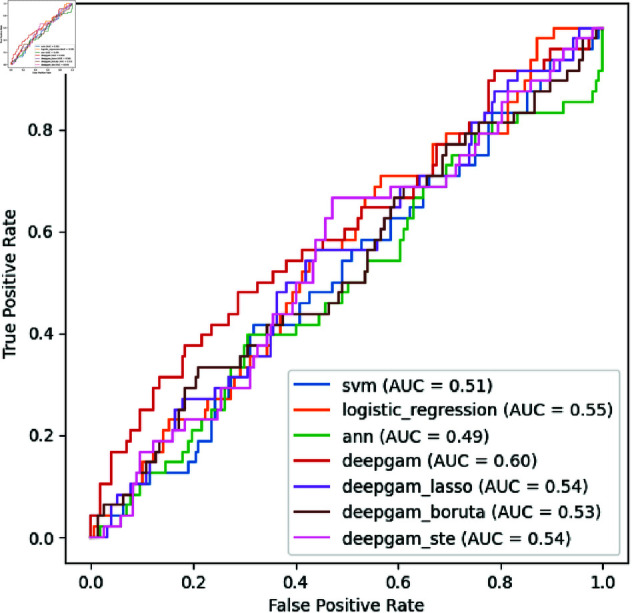
ROC curve visualization.

Table 1 demonstrates the performance comparisons for depression diagnosis in the validation (test) set. We compared DeepGAM’s performance with traditional machine learning models and IGANN, a GAM-based interpretable model. DeepGAM, utilizing all 99 features, demonstrated superior performance across most evaluation metrics. Specifically, it achieved the highest scores in AUC of 0.600, F1-score of 0.387, and Precision of 0.324, outperforming all compared methods. Especially, the AUC of DeepGAM was 0.045 higher than the second-best method (Logistic Regression), where Fig 2 visualizes the performance gap in ROC curves (red curve *vs.* orange curve). Table 2 illustrates the outstanding performance of DeepGAM in 5-fold cross-validation. These results indicate that the additive functions in DeepGAM achieve better representation learning for depression diagnosis.

**Table 1:** Validation performance comparisons with machine learning and feature selection methods for depression estimation. The bold entries represent the best performance for the same number of features (99 and 5).

Method	#features	AUC	Accuracy	F1-score	Precision	Recall	Specificity
SVM	99	0.514	0.556	0.355	0.269	0.521	0.567
Logistic Regression	99	0.555	0.532	0.377	0.274	**0.604**	0.510
Neural Network	99	0.492	0.546	0.340	0.258	0.500	0.561
IGANN	99	0.516	**0.746**	0.212	0.146	0.389	**0.930**
DeepGAM (Ours)	99	**0.600**	0.644	**0.387**	**0.324**	0.479	0.694
DeepGAM + Unimportant features (random)	5	0.539	**0.533**	0.289	**0.274**	0.468	0.533
DeepGAM + Lasso	5	0.544	0.468	0.363	0.252	0.646	0.414
DeepGAM + Boruta	5	0.527	0.478	0.327	0.234	0.542	**0.459**
DeepGAM + STE (Ours)	5	**0.545**	0.502	**0.386**	0.271	**0.667**	0.452

**Table 2:** Cross-validation performance comparisons with machine-learning methods for depression estimation.

Method	#features	AUC	Accuracy	F1-score	Precision	Recall	Specificity
SVM	99	0.470	0.572	0.415	0.306	0.676	0.545
SVM + STE	5	0.437	0.580	0.403	0.297	0.643	0.563
Logistic Regression	99	0.508	0.577	0.395	0.295	0.614	0.568
Logistic Regression + STE	5	0.498	0.547	0.369	0.271	0.598	0.528
Neural Network	99	0.459	0.541	0.397	0.286	0.667	0.500
Neural Network + STE	5	0.454	0.487	0.405	0.281	0.776	0.401
DeepGAM (Ours)	99	0.600	0.588	0.411	0.311	0.637	0.573
DeepGAM + STE (Ours)	5	0.589	0.567	0.403	0.295	0.661	0.534

#### Feature selection performance comparisons.

Table 1 also presents the performance comparisons with feature selection methods, which select the top 5 most important features from 99 features. The experimental results showed a significant decline in performance when using Lasso and Boruta for feature selection compared to the baseline model with all 99 features. Specifically, reducing the number of features to 5 with Lasso and Boruta resulted in a considerable drop in F1-scores. However, the STE method demonstrated superior performance in feature selection; DeepGAM + STE achieves better scores in most measures than DeepGAM+Lasso and DeepGAM+Boruta. DeepGAM + STE performs similar F1-scores and achieves higher Recall compared to DeepGAM. Fig 2 demonstrates that the DeepGAM + STE outperforms all compared methods around 0.5 False Positive Rate. Moreover, STE maintains the model performance for SVM, Logistic Regression, Neural Network, and DeepGAM in 5-fold cross-validation as visualized in Table 2. This indicates that STE effectively identifies the most relevant features, which helps preserve or enhance the model’s capabilities even with fewer features.

Table 1 also presents the performance results when using five features randomly selected from the remaining features not selected by the STE, Lasso, or Boruta methods, denoted as DeepGAM + Unimportant features. The reported performances are the average values obtained by randomly selecting five features 10 times. DeepGAM + Unimportant features underperformed DeepGAM + STE in AUC (0.539 *vs.* 0.545), F1-score (0.289 *vs.* 0.386), and Recall (0.468 *vs.* 0.667). These results highlight the effectiveness of the STE method in selecting important features.

#### Important feature comparisons.

Table 3 compares the top 5 features identified as most important by three different feature selection methods: Lasso, Boruta, and STE (our proposed method). Each method aims to identify the most relevant features that contribute significantly to the model’s predictive power. This comparison illustrates how different feature selection methods can yield different sets of important features; only two features (Osteopontin and Creatinine, Urine, Total) overlap through the three methods. As we discussed in the above section, the choice of method can significantly impact the model’s performance and interpretability. In this case, while Lasso and Boruta provided valuable insights, the STE method showed a more effective selection of features that preserved or improved the model’s AUC, Accuracy, F1-score, Precision, Recall, and Specificity.

**Table 3:** Top 5 important features selected by feature selection methods.

Method	Top 5 important features				
Lasso	Leptin	Resistin	C20 3n6 Fatty Acid	Creatinine, Urine, Total	Calcium
Boruta	Osteopontin	Creatinine, Urine, Total	Vitamin DBP	MCV	Myeloperoxidase
STE (Ours)	TNF alpha	Osteopontin	Plasma NGAL	Microalbumin:Creatinine Ratio	NT-PROBNP

#### Interpretability visualization.

**Fig 3:**
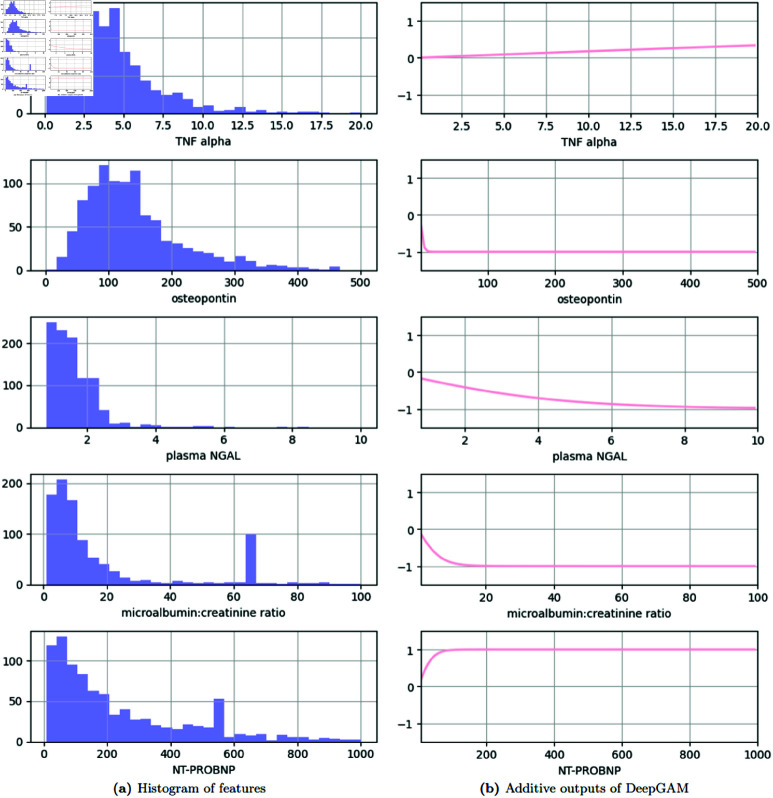
Effect visualization of each feature. (Left) Distribution histogram of each feature. (Right) The output of the additive function in (Eq 5) has the range of –1 to +1 for each feature value.

Fig 3 presents the distributions (left) and predicted trends from DeepGAM + STE (right) of the top 5 important features selected by the STE method, providing valuable insights from the perspective of interpretability. It highlights the unique patterns of each feature in terms of data distribution and model predictions, enabling a visual understanding of how the model responds to the data. TNF alpha values are mostly concentrated between 2 and 10, peaking around 4-6. The predicted trend indicates a relatively flat response, suggesting that changes in TNF alpha have minimal impact on the model’s output within the observed range. Osteopontin values are predominantly below 200, with a significant peak between 50 and 150. The trend shows a steep decline initially, which quickly levels off. Plasma NGAL values are heavily skewed to the right, with most values falling between 0 and 2. Plasma NGAL shows a relatively flat response to negative. Microalbumin: Creatinine Ratio has a pronounced right skew, with most values clustered below 20. The trend for this ratio indicates a steep decline. NT-PROBNP values show a right-skewed distribution, with most observations below 200. The predicted trend shows a steep incline initially. The histograms demonstrate that all selected features have right-skewed distributions, indicating that higher values are less frequent. The predicted trends suggest that for each feature, lower values generally contribute less impact on (anti-)depression, with a positive or negative impact on high values. According to Fig 3, all selected features exhibit right-skewed distributions, with high values occurring less frequently. The analysis of predicted trends reveals that lower feature values have relatively less impact on predictions, while higher values show an increasing tendency to have either positive or negative effects.

**Fig 4:**
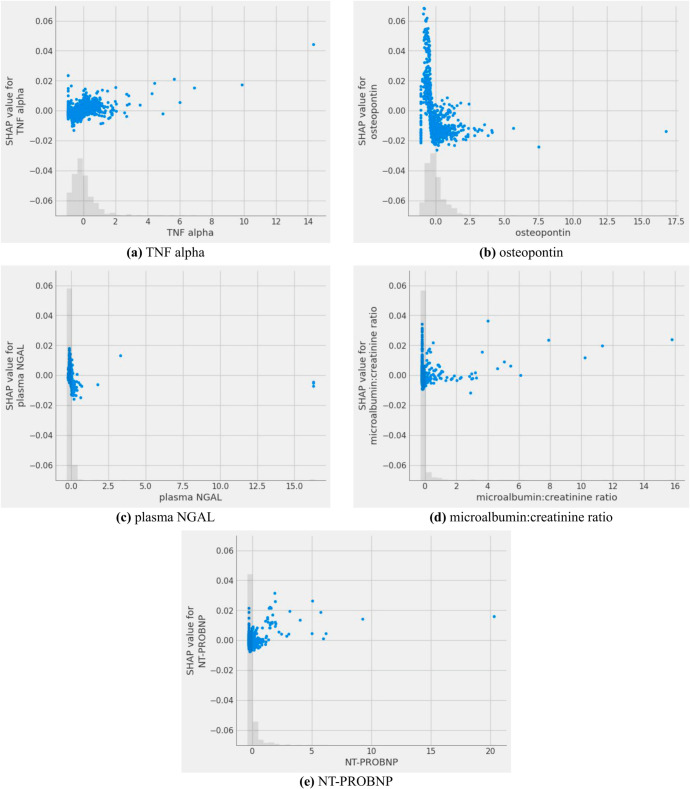
SHAP-based visualization for feature-wise contribution comparison.

To further validate the impact of the five key features selected by the Straight-Through Estimator (STE) method on depression prediction, Fig 4 presents an interpretability analysis using SHapley Additive exPlanations (SHAP). The data distributions of all five features were similarly centered around zero in both methods. Regarding trend consistency, SHAP showed similar patterns to DeepGAM + STE for TNF alpha, osteopontin, plasma NGAL, and NT-PROBNP. However, a noticeable difference was observed in the microalbumin: creatinine ratio. While DeepGAM + STE indicated a substantial negative contribution at low values, SHAP results showed minimal local contributions across most data points. This discrepancy stems from the inherent difference in interpretability mechanisms: SHAP explains the influence of each feature locally on a per-sample basis, whereas DeepGAM + STE learns the global functional relationship between input features and model output across the entire distribution.

### Analysis

#### Ablation study.

**Fig 5:**
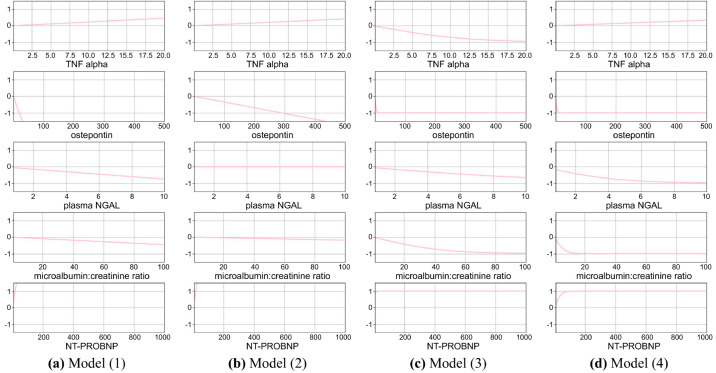
Additive output visualization of ablation study in Table 4.

Table 4 presents an ablation study of DeepGAM, evaluating the impact of different components on performance metrics. The study examined four configurations of the model by including or excluding the interpretability regularizer (${ℛ}_{1}$) and the Tanh activation function in (Eq 5). DeepGAM (Model (4)) achieved the highest AUC and F1-score, where ${ℛ}_{1}$ and tanh activation restricted the model capabilities. This indicates that the restriction leads to learning better representation. Fig 5 visualizes the additive outputs for interpretability from the models described in Table 4, illustrating the impact of individual features on predictions. Model (1), without any regularization or activation function, generally shows a simple downward or upward trend over –1 or +1 for most features. Model (2), with only the interpretability regularizer, shows each feature’s reduced impact (low magnitude). Model (3), with only the tanh activation, shows a bounded impact between –1 and +1, with notable increases in Recall and decreases in Specificity. Method (4), which includes both the interpretability regularizer and the tanh activation, achieves the best balance across all metrics, suggesting that combining these two components enhances model performance and interpretability.

**Table 4:** Ablation study of DeepGAM.

Model	Interpretability regularizer (${ℛ}_{1}$)	tanh activation Eq 5	AUC	Accuracy	F1-score	Precision	Recall	Specificity
(1)	✘	✘	0.524	0.459	0.368	0.252	0.667	0.395
(2)	✓	✘	0.527	0.283	0.369	0.232	**0.896**	0.096
(3)	✘	✓	0.511	**0.580**	0.358	**0.279**	0.500	**0.605**
(4)	✓	✓	**0.545**	0.502	**0.386**	0.271	0.667	0.452

#### Selected features during training.

**Fig 6:**
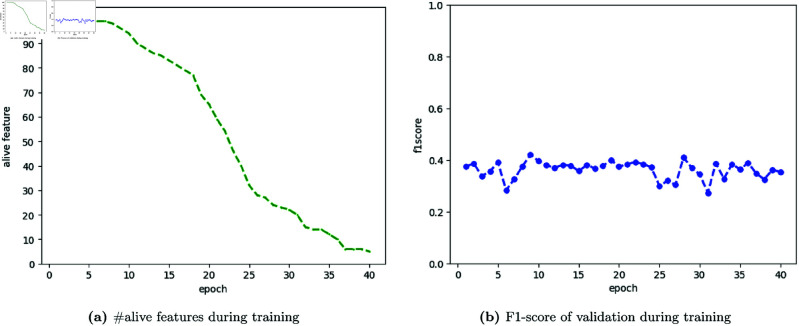
Visualization of feature selection during training. ( **a**) Straight-Through Estimator (STE) *deactivates* each feature during training. ( **b**) The prediction model maintains the F1-scores with a reduced number of features.

Fig 6 illustrates the feature selection process during training using the Straight-Through Estimator (STE) method. Fig 6a shows the number of active features decreasing from 99 to 5 over 40 epochs, indicating STE’s effectiveness in deactivating less important features. Fig 6b demonstrates that the F1-score remains stable throughout the training despite reducing the number of active features. This stability suggests that the model maintains its performance by focusing on the most relevant features, highlighting STE’s ability to simplify the model without compromising predictive power.

#### Model performance comparisons on public datasets.

To evaluate the generalizability of DeepGAM, we conducted additional experiments on three publicly available benchmark datasets.

First, Table 5 presents performance comparisons on the breast cancer dataset provided by scikit-learn. In this dataset, the AUC and F1-scores of SVM and Logistic Regression were 0.9951 and 0.9645, respectively, whereas DeepGAM achieved higher scores of 0.9971 and 0.9722. Compared to a Neural Network, DeepGAM showed slightly lower results in specific metrics like Precision and Specificity; however, it holds significance in terms of interpretability. When comparing DeepGAM with IGANN, another interpretable model, DeepGAM achieved a higher AUC of 0.9971 than IGANN’s 0.9967. Thus, DeepGAM performs comparably to standard machine learning models and provides interpretability, demonstrating its accurate performance and interpretability on the breast cancer dataset.

**Table 5:** Performance comparisons on public data (cancer).

Method	AUC	Accuracy	F1-score	Precision	Recall	Specificity
SVM	0.9951	0.9561	0.9645	0.9714	0.9577	0.9535
Logistic Regression	0.9951	0.9561	0.9645	0.9714	0.9577	0.9535
Neural Network	0.9980	0.9649	0.9726	0.9467	1.000	0.907
IGANN	0.9967	0.9737	0.9790	0.9722	0.9859	0.9535
DeepGAM (Ours)	0.9971	0.9649	0.9722	0.9589	0.9859	0.9302

Second, Table 6 compares model performances on another depression dataset provided by Kaggle (https://www.kaggle.com/datasets/diegobabativa/depression). DeepGAM outperformed SVM with a higher F1-score, Precision, and Recall, scoring 0.2458, 0.2458, and 0.4681, respectively, versus SVM’s scores of 0.2168, 0.1545, and 0.3617. Compared with a Neural Network, DeepGAM achieved better Accuracy and Specificity, with values of 0.5280 and 0.5397, respectively, versus 0.4056 and 0.5397 for the Neural Network. Although DeepGAM showed slightly lower performance than Logistic Regression, its interpretability adds value. When compared to IGANN, another interpretable model, DeepGAM exhibited better AUC, F1-score, and Recall, with values of 0.5140, 0.2458, and 0.4681, compared to IGANN’s 0.4903, 0.2353, and 0.3404.

**Table 6:** Performance comparisons on public data (depression).

Method	AUC	Accuracy	F1-score	Precision	Recall	Specificity
SVM	0.5328	0.5699	0.2168	0.1545	0.3617	0.6109
Logistic Regression	0.5585	0.5804	0.3103	0.2126	0.5745	0.5816
Neural Network	0.5297	0.4056	0.2672	0.1676	0.6596	0.3556
IGANN	0.4903	0.6364	0.2353	0.1798	0.3404	0.6946
DeepGAM (Ours)	0.5140	0.5280	0.2458	0.1667	0.4681	0.5397

Third, Table 7 compares model performances on the diabetes dataset provided by scikit-learn, using MSE and ${\text{R}}^{2}$ as evaluation metrics for this regression task. DeepGAM performed lower than other models on this dataset, as it is designed for classification tasks. IGANN, which is designed for regression and classification tasks, performed better on this dataset, indicating DeepGAM’s limitation in handling regression problems.

**Table 7:** Performance comparisons on public data (diabetes).

Method	MSE	R^2^ score
SVM	18754.3867	–2.5398
Neural Network	2894.0762	0.4538
IGANN	2886.1699	0.5133
DeepGAM (Ours)	3165.0042	0.1887

Overall, the results indicate that DeepGAM maintains competitive performance compared to standard machine learning models (SVM, Logistic Regression, and Neural Network) and an interpretable model (IGANN) for classification tasks, providing accurate prediction and interpretability. However, DeepGAM shows some limitations in regression tasks. Future improvements could enhance DeepGAM’s performance and interpretability in regression tasks, enabling broader applicability.

## Conclusion

We introduced DeepGAM, an interpretable deep neural network framework for diagnosing depression using personal medical data. DeepGAM leverages the Generalized Additive Model (GAM) to enhance interpretability and integrates a Straight-Through Estimator (STE) for effective feature selection. Additionally, DeepGAM incorporates an interpretability regularizer and a tanh activation function to improve model transparency and diagnostic capability. Experimental results demonstrated that DeepGAM outperformed traditional machine learning models across various performance metrics, and STE allowed the model to maintain high performance with fewer features. Furthermore, DeepGAM performed better than IGANN, another GAM-based interpretable model for classification tasks. However, DeepGAM showed performance limitations in regression tasks due to the use of the tanh activation function and regularizer, which impose hard and soft constraints on prediction values.

The Heart and Soul Study provides valuable information on the relationship between psychological and cardiovascular health; however, several limitations exist. The study cohort is predominantly older, male, and non-Hispanic white, limiting the generalizability of the findings to broader and more diverse populations. Selection bias is also a concern, as participants were specifically chosen for stable coronary heart disease, excluding individuals with recent myocardial infarction or severe conditions, potentially skewing results towards healthier individuals. Additionally, reliance on self-reported measures, such as physical activity and psychological factors, introduces potential measurement bias, as these reports may be inaccurate or influenced by subjective interpretation. Confounding factors, such as socioeconomic status or access to healthcare, may also affect outcomes and are challenging to account for in analyses fully. Finally, longitudinal follow-up introduces the risk of survivor bias, as individuals lost to follow-up or who passed away early are excluded from long-term observations. These limitations highlight the need for caution in interpreting results and underscore the importance of validating findings in more diverse and representative populations.

In future research, several specific approaches could enhance predictive accuracy. First, incorporating a multimodal approach that integrates diverse data types—such as clinical records, genetic information, imaging data (e.g., MRI or CT scans), and patient-reported outcomes—could substantially improve predictive accuracy. For example, combining genomic information with medical imaging may enable a more precise analysis of disease risk factors. Second, incorporating factors not captured in the Heart and Soul Study, such as lifestyle elements (e.g., diet or sleep patterns) and environmental aspects (e.g., air quality or social environment), could facilitate the development of more sophisticated models for predicting disease onset. Third, the real-time collection and analysis of physiological signals, such as heart rate, blood pressure, and body temperature, could enable faster detection and prediction of disease development. Fourth, assessing a patient’s overall health by including emotional state and mental health data could be valuable for predicting mental health disorders. Finally, considering social factors, such as socioeconomic status or levels of social support, may also play a critical role in improving model performance. By leveraging these additional features, predictive models can be further strengthened.
